# Local and Remote Postconditioning Decrease Intestinal Injury in a Rabbit Ischemia/Reperfusion Model

**DOI:** 10.1155/2016/2604032

**Published:** 2015-12-27

**Authors:** Mu Yang, Jian-Xin Dong, Lu-Bin Li, Hai-Jie Che, Jun Yong, Fu-Bo Song, Tao Wang, Jv-Wen Zhang

**Affiliations:** Department of Vascular Surgery, Yantai Yuhuangding Hospital, The Affiliated Hospital of Medical College, Qingdao University, Yantai 264000, China

## Abstract

Intestinal ischemia/reperfusion (I/R) injury is a significant problem that is associated with high morbidity and mortality in critical settings. This injury may be ameliorated using postconditioning protocol. In our study, we created a rabbit intestinal I/R injury model to analyze the effects of local ischemia postconditioning (LIPo) and remote ischemia postconditioning (RIPo) on intestinal I/R injury. We concluded that LIPo affords protection in intestinal I/R injury in a comparable fashion with RIPo by decreasing oxidative stress, neutrophil activation, and apoptosis.

## 1. Introduction

Acute mesenteric ischemia (AMI) is an acute disorder of bowel circulation caused by mesenteric arterial embolism or thrombosis [1]. Ischemic flow of the bowel can be restored rapidly with the clinical application of thrombolysis and thrombectomy; however, injury induced by intestinal ischemia/reperfusion (I/R) remains problematic. Parks and Granger [2] showed that the injury caused by I/R could be more severe than that of ischemia alone. Injuries sustained from I/R can be particularly damaging to the small intestine [3]. I/R can not only cause intestinal tissue damage, but also induce systemic circulation of intestinal bacteria or endogenous endotoxins, which can lead to systemic multiorgan dysfunction syndrome (MODS) [4]. Therefore, it is important to attenuate intestinal I/R injury.

Previously, Murry et al. found that ischemic preconditioning (IPC), a process consisting of repetitive brief episodes of ischemia, rendered the myocardium more resistant to prolonged ischemic insult and irreversible injury [5]. Although several studies have shown that IPC decreased intestinal I/R injury in I/R animal models, the major barrier to the use of IPC in treating I/R in clinical practice is due to the unpredictable onset of I/R. Zhao et al. proposed the concept of local ischemia postconditioning (LIPo), which consists in performing one or more short cycles of intermittent reperfusion applied immediately after an ischemic phase and before the permanent reperfusion occurs [6]. This has been shown to exhibit a protective effect against I/R injuries. While the protective effects of LIPo on reperfusion injury have been established in animal organs, including heart [6], brain [7], kidney [8], and skeletal muscle [9], the effects of LIPo on mesenteric ischemia have not been elucidated.

Kerendi et al. [10] proposed the concept of remote ischemic postconditioning (RIPo) as an endogenous phenomenon in which the application of one or more brief cycles of nonlethal ischemia and reperfusion to an organ or tissue protects a remote organ or tissue from a sustained episode of lethal I/R injuries. However, the mechanism by which the protective effect of RIPo occurs has not been studied in small intestinal I/R injury.

The hypothesis of the current study is that LIPo and RIPo can protect against intestinal I/R injury by decreasing oxidative stress, neutrophil activation, and apoptosis.

## 2. Materials and Methods

### 2.1. Animals

Twenty-four male New Zealand White rabbits weighing between 2.0 and 2.5 kg were obtained from the Animal Research Center at Binzhou Medical School, Shandong, China. All animals used in this study were handled according to* The Guide for the Care and Use of Laboratory Animals* published by the United States National Institute of Health (NIH publication number 85-23, revised 1996). All procedures in this study were approved by the Committee of Experimental Animals of Qingdao University, Shandong, China. The rabbits were housed in individual cages in a temperature-controlled room and acclimated for 1 week before initiation of experiments. Food was removed 8 h prior to the study, but all animals had free access to water.

### 2.2. Establishment of the Intestinal I/R Model

Animals were anesthetized with sodium pentobarbital, 35 mg/kg, i.p. Rabbits were secured in a supine position on a heating pad to maintain body temperature. The small intestine was exteriorized by midline laparotomy, and superior mesenteric artery (SMA) and the left femoral artery (LFA) were exposed. Intestinal I/R injury was established by occluding the SMA for 30 min with an atraumatic arterial clamp, followed by reperfusion for 120 min.

### 2.3. Sample Preparation

**Figure 1:**
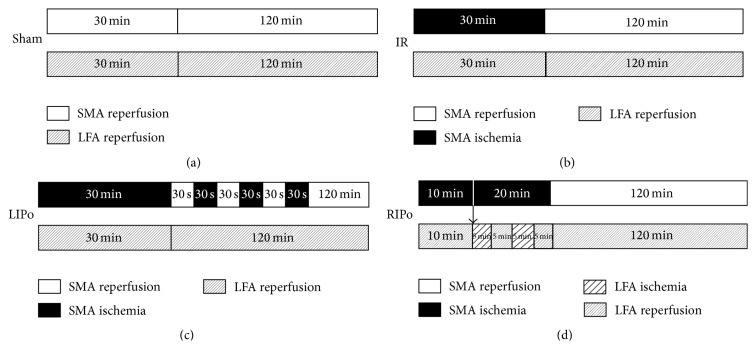
Experimental protocol. (a) Sham: laparotomy exposing the SMA and LFA; (b) I/R: 30 min of SMA occlusion followed by 120 min reperfusion with the LFA intact; (c) LIPo: 3 cycles of 30 s reperfusion-reocclusion of SMA were applied at the onset of SMA reperfusion; (d) RIPo: 2 cycles of 5 min reperfusion-reocclusion of the LFA were applied when SMA was occluded for 10 min and at the time of SMA reperfusion.

Subjects were randomly allocated into four groups (*n* = 6) as shown in Figure 1: (a) sham (laparotomy exposing the SMA and LFA), (b) I/R (30 min of SMA occlusion followed by 120 min reperfusion with the LFA intact), (c) LIPo (after induction of 30 min ischemia, 3 cycles of 30 s reperfusion-reocclusion of SMA were applied at the onset of reperfusion), and (d) RIPo (2 cycles of 5 min reperfusion-reocclusion of the LFA were applied when SMA was occluded for 10 min and at the time of SMA reperfusion). After 120 min of reperfusion, 5 mL blood samples were collected from the inferior vena cava and stored at 4°C for 5 h. Serum was isolated by centrifugation at 4000 r/min for 5 min and stored at −80°C for malondialdehyde (MDA) measurements [11]. Two segments of 5 cm of the intestine were harvested from the ileum. One portion of intestinal sample was washed in cold phosphate-buffered saline, snap-frozen in liquid nitrogen, and then stored at −80°C for further study. Another segment was fixed in 4% formaldehyde and paraffin and embedded for intestine mucosal morphological and apoptosis analysis.

### 2.4. Evaluation of Intestinal Mucosal Histopathology

A portion of the small intestine was stained with hematoxylin and eosin (H&E). Intestinal mucosal damage was evaluated independently by two pathologists who were blinded to the study. The degree of injury was evaluated based on changes of the villi and glands of the intestinal mucosa (a modified method from Chiu et al.) [12]. The damage was graded from 0 (normal) to 5 (severely damaged) as follows: grade 0, normal mucosal villi; grade 1, development of subepithelial Gruenhagen's space at the apex of the villus, often associated with capillary congestion; grade 2, extension of the subepithelial space with moderate lifting of the epithelial layer from the lamina propria; grade 3, massive epithelial lifting down the sides of villi, possibly with a few denuded tips; grade 4, denuded villi with lamina propria and dilated capillaries exposed, possibly with increased cellularity of lamina propria; and grade 5, digestion and disintegration of the lamina propria, hemorrhage, and ulceration. A minimum of 5 randomly chosen fields from each sample were evaluated and averaged to determine the extent of mucosal damage.

### 2.5. Measurement of MDA in Serum and Intestinal Tissues

MDA is a direct product of lipid peroxidation and reflects the extent of lipid peroxidation. Intestinal tissues were weighed and then processed into a 10% homogenate. MDA activity (expressed as nmol/mL or nmol/mg) in the serum and intestinal tissues was determined using a chemical assay kit (Nanjing Jiancheng Bioengineering, Nanjing, China), measuring thiobarbituric acid reactive substance levels [11].

### 2.6. Measurement of Myeloperoxidase (MPO) and Superoxide Dismutase (SOD) in Intestinal Tissues

Intestinal tissues were processed into 5% and 1% homogenates for MPO measurements, respectively. The enzymatic activities of MPO in intestinal tissues were measured with commercial kits (Nanjing Jiancheng Bioengineering, Nanjing, China). MPO activity was used to assess neutrophil infiltration in the intestine, which is a sensitive measure of damage to the intestinal mucosal barrier [13]. MPO activity was calculated as the amount of tissue capable of degrading 1 *μ*mol peroxide/1 min at 37°C and was expressed as U/g in the tissue. Intestinal mucosal tissues were homogenized on ice with normal saline, frozen at −20°C for 5 min, and centrifuged for 15 min at 4,000 ×g. Supernatants were transferred into fresh tubes for evaluation. The lipid peroxidation products of SOD activity were measured using chemical assay kits (Nanjing Jiancheng Biological Product, Nanjing, China). The results were expressed as U/mg protein.

### 2.7. Evaluation of Intestinal Mucosal Epithelial Apoptosis

Analysis of intestinal mucosal epithelial cell apoptosis was carried out by the TdT- (terminal deoxynucleotidyl transferase-) mediated dUDP-biotin nick-end labeling (TUNEL) [14]. Cell death was assessed using an* in situ* detection assay kit (Roche, Indianapolis, IN). TUNEL-positive cells were characterized by brown staining of the nucleus and nuclear membrane. Quantitation of apoptotic cells was performed independently by two pathologists who were blinded to the study groups. The number of positive cells in three randomly chosen fields within each slide was manually counted at an original magnification of 200x. The rate of cell apoptosis (apoptotic index) was expressed as a percentage of TUNEL-positive cells using the following formula: number of TUNEL-positive cell nuclei/the number of total cell nuclei × 100.

### 2.8. Statistical Analysis

All data were expressed as means ± standard error of means. Statistical analysis was performed by analysis of variance (ANOVA) followed by Tukey's post hoc test. The null hypothesis was rejected at the 0.05 level of significance. SPSS11.0 software (Chicago, IL, USA) was used for data analysis.

## 3. Result 

### 3.1. Intestinal Histopathological Damage Was Decreased by LIPo or RIPo

**Figure 2:**
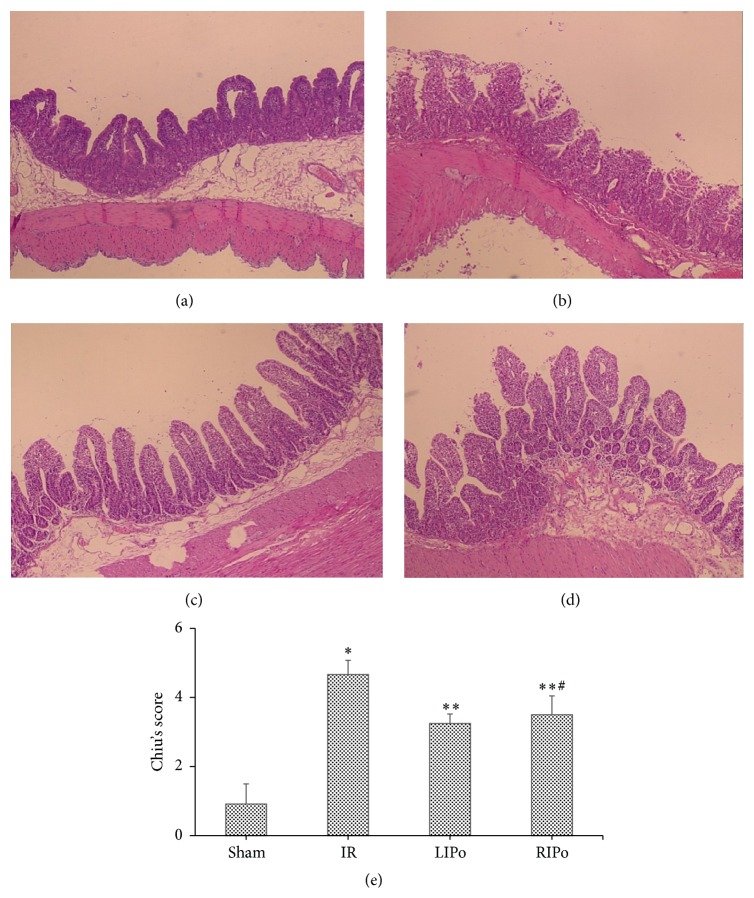
Histopathological changes of the intestinal mucosa and the evaluation of intestinal injury by Chiu scores (HE staining, magnification 200x). (a) Sham group. (b) I/R group. ((c) and (d)) LIPo and RIPo groups. (e) Changes of the intestinal mucosa Chiu scores. Data are presented as mean ± SD, *n* = 6. ^*∗*^ *p* < 0.05 versus the sham group; ^*∗∗*^ *p* < 0.05 versus the I/R group; ^#^ *p* > 0.05 versus the LIPo group.

Representative photomicrographs of hematoxylin and eosin (H&E) stained intestinal samples are shown in Figures 2(a)–2(d). The intestinal mucosal injury was evaluated using Chiu's scores (Figure 2(e)). Injury scores of the I/R group (4.67 ± 0.17) were significantly higher than those of the sham group (0.92 ± 0.58) (*p* < 0.05). Injury scores of the sham group were also significantly lower than those of the LIPo (3.25 ± 0.27) or the RIPo group (3.50 ± 0.55) (*p* < 0.05 versus I/R group). There was no significant difference between the injury scores of the LIPo (3.25 ± 0.27) and the RIPo (3.50 ± 0.55) groups (*p* > 0.05).

### 3.2. The Levels of Serum and Intestinal MDA Were Decreased in the LIPo and RIPo Groups

**Figure 3:**
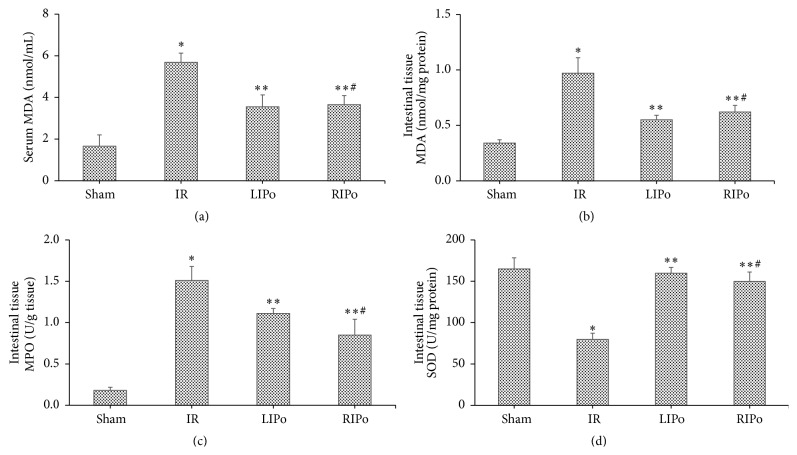
Effects of LIPo and RIPo on MDA, MPO, and SOD levels. Serum MDA (a), intestine MDA (b), MPO (c), and SOD activity (d). Data are mean ± SD, *n* = 6. ^*∗*^ *p* < 0.05 versus the sham group; ^*∗∗*^ *p* < 0.05 versus the I/R group; ^#^ *p* > 0.05 versus the LIPo group ((a), (b), and (d)); ^#^ *p* < 0.05 versus the LIPo group (c).

Serum MDA activity of the I/R group (6.69 ± 0.45) was higher compared to that of the sham group (1.68 ± 0.45) (*p* < 0.05) and lower compared to both LIPo (3.58 ± 0.61) and RIPo groups (3.66 ± 0.44) (*p* < 0.05 versus I/R group) (Figure 3(a)). There was no significant difference in the serum MDA activity between the LIPo (3.58 ± 0.61) and the RIPo groups (3.66 ± 0.44) (*p* > 0.05). Similarly, MDA activity of the I/R group (0.98 ± 0.14) was higher compared to that of the sham group (0.34 ± 0.03) (*p* < 0.05) but lower compared to that of the LIPo (0.57 ± 0.05) and the RIPo groups (0.62 ± 0.06; *p* < 0.05 versus I/R group; Figure 3(b)). There was no significant difference in MDA activity between the LIPo (0.57 ± 0.05) and the RIPo groups (0.62 ± 0.06) (*p* > 0.05).

Levels of MPO were decreased in intestinal tissues of the LIPo and RIPo groups. Intestinal tissue MPO activity was higher in the I/R group (1.51 ± 0.17) compared to that in the sham group (0.17 ± 0.04) (*p* < 0.05), and it was lower compared to both the LIPo (1.13 ± 0.10) and the RIPo groups (0.85 ± 0.19) (*p* < 0.05 versus IR group; Figure 3(c)). Intestinal tissue MPO activity of the LIPo group was significantly higher than that of the RIPo group (0.85 ± 0.19; *p* < 0.05).

Levels of SOD were increased in intestinal tissues by LIPo and RIPo. Intestinal tissue SOD activity of the I/R group (79.62 ± 7.60) was significantly lower than that of the sham group (165.00 ± 13.19) (*p* < 0.05) (Figure 3(d)), but it was higher than that of the LIPo (149.70 ± 21.30) and RIPo groups (149.80 ± 11.29) (*p* < 0.05 versus IR group). There was no significant difference in SOD activity between the LIPo (149.70 ± 21.30) and the RIPo groups (149.80 ± 11.29) (*p* > 0.05).

### 3.3. LIPo and RIPo Reduced the Apoptotic Rate after I/R Injury

**Figure 4:**
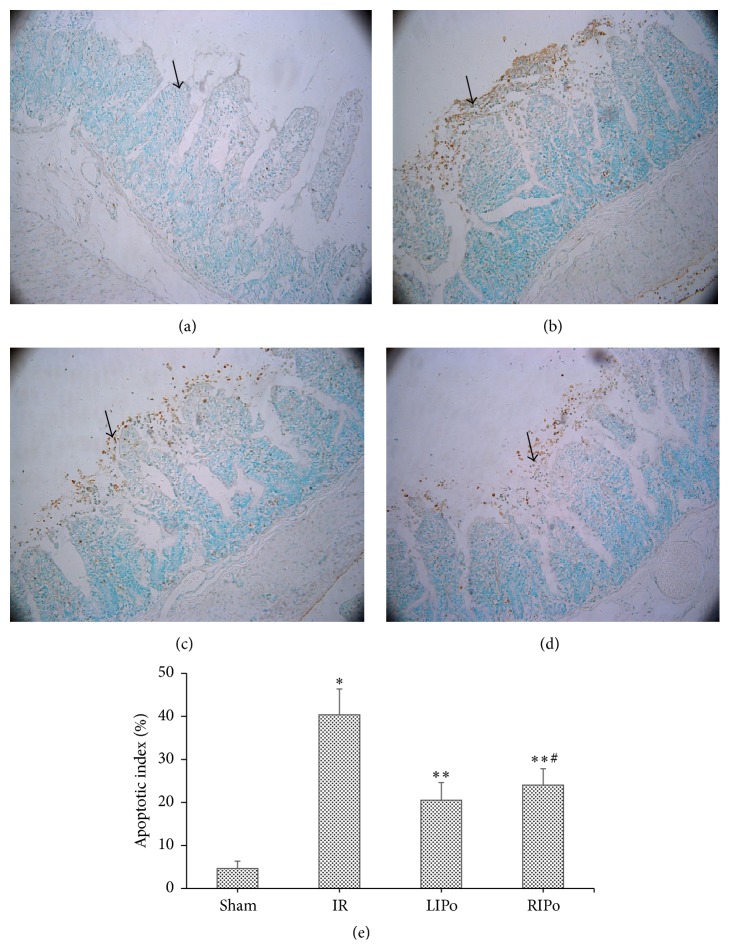
(e) Apoptotic indexes of each group (magnification 200x). Data were represented as mean ± SD, *n* = 6. ^*∗*^ *p* < 0.05 versus the sham group; ^*∗∗*^ *p* < 0.05 versus the I/R group; ^#^ *p* > 0.05 versus the LIPo group.

Intestinal mucosal epithelial apoptosis detected by TUNEL assay is shown in Figures 4(a)–4(d) and was quantified as apoptotic index (Figure 4(e)). The apoptotic index of the I/R group (40.35 ± 6.03) was significantly higher than that of the sham group (4.65 ± 1.79) (*p* < 0.05) and LIPo (23.35 ± 5.00) and RIPo groups (20.55 ± 4.03) (*p* < 0.05 versus I/R group). There was no significant difference in the apoptotic index between the LIPo (23.35 ± 5.00) and the RIPo groups (20.55 ± 4.03) (*p* > 0.05).

## 4. Discussion

A number of studies have suggested potential techniques to reduce the impact of ischemia/reperfusion injury. Hotter et al. showed that brief 10 min ischemia followed by 5 min reperfusion decreased intestinal injuries caused by intestinal reperfusion in rats [15]. Sola et al. [16] and others [17] showed that IPC exhibits protective effects on intestinal ischemia/reperfusion injury. dos Santos et al. found that LIPo produced similar effects to IPC in preventing intestinal I/R injuries [12]. They established a rat model of mesenteric artery ischemia consisting of 30 min occlusion followed by 60 min reperfusion. Postconditioning using 3 cycles of 2 min reperfusion and 2 min reocclusion effectively decreased mucosal damage. Similar results were obtained by postconditioning with prolonged 60 min ischemia and 3 cycles of 30 sec reperfusion and reocclusion [18]. Studies using other animal models showed that LIPo before reperfusion mitigated oxidative stress and histologic damage during porcine small bowel autotransplantation [19]. However, Bretz et al. showed that, after 45 min of complete vascular occlusion of the jejunum, postconditioning with 4 cycles of 30 sec reperfusion and 30 sec reocclusion during the initial 4 min of 2 h reperfusion did not decrease the effects of prolonged I/R on rabbit small intestine [20]. We used a similar rabbit model but obtained different results.

In our current study, LIPo significantly reduced histological damage of rabbit intestines in the I/R and LIPo groups. To date, there have been no studies on the effects of LIPo among different organs in different species. However, there have been several studies that failed to demonstrate a positive effect of LIPo on reducing intestinal damage. It is possible that these results were derived from differences in model establishment, methods of postconditioning, or duration of LIPo adaptation. In our current study, we used a modified protocol from Bretz et al. [20], in which LIPo was introduced immediately after I/R, and 3 cycles of 30 sec reperfusion and reocclusion were applied. The main difference between the two studies is that, in our intestinal model, I/R injury was induced by 30 min of ischemia and 120 min of reperfusion of the superior mesenteric artery, whereas 45 min ischemia to the jejunal arteries was used in the study by Bretz et al. Liu et al. suggested that the greater the intestinal damage, the better the model to observe the protective effect of LIPo, supporting the notion that our current intestinal model might be more suitable to study the protective effect of LIPo. In addition, we explored the mechanism of I/R and found that oxidative stress, activation of neutrophil, and apoptosis play significant roles in the pathogenesis of intestinal I/R injury.

Although IPo can be applied after ischemia, it has to be carried out at the original location where ischemia occurred. Therefore, it can be difficult to control the degree of ischemic postconditioning in ischemia-sensitive organs. Previous studies have examined whether IPo can be applied at distal region and showed that single 5 min occlusion and reperfusion at the renal artery significantly reduced myocardial ischemia/reperfusion injury in a rat coronary occlusion-reperfusion model [10]. The other advantage of RIPo* in situ* is that RIPo can be performed noninvasively, as the reperfusion and occlusion procedure can be performed by placing a tourniquet around the proximal part of a limb. Andreka et al. performed RIPo with 4 cycles of 5 min occlusion pressure applied to the lower limb immediately after reperfusion and showed its protective effect on the heart during acute myocardial infarction in pigs [21]. In the current study, using intestinal pathological scores, we found that RIPo protects the intestine from IR injury. However, whether IPo or RIPo is a better protective technique remains controversial. Gritsopoulos et al. [22] demonstrated that RIPo is more potent than classical IPo in reducing the infarct size in anesthetized rabbits. On the other hand, our study showed no difference between RIPo and LIPo, as there is no significant difference found in pathological grading, apoptosis index, or MDA/SOD, except that MPO in the RIPo group was lower than that of the LIPo group. Study by Kadkhodaee et al. [23] also found no difference between the two adaptations, as 4 cycles of 5 min I/R of left femoral artery at the onset of reperfusion of previously ischemic kidneys had no additional benefit in the reduction of blood urea and creatinine when compared to LIPo. Dissimilarity in different species and organs, variations of the site where RIPo was performed, and the algorithm of experimental design can all contribute to the discrepancy between studies.

Intestinal I/R injury is a complex process that involves multiple pathophysiologic mechanisms. It has been shown that ROS are released immediately after the onset of reperfusion, causing release of a large amount of ROS, including super oxygen, hydrogen peroxide, and hydroxy radical [24, 25]. Excessive levels of free radicals may lead to lipid peroxidation and induction of damage to the cell membranes and mitochondria. Neutrophil activation and release of inflammatory cytokines [26] eventually cause cell apoptosis and necrosis. The current study showed decreased pathological scores, apoptosis indexes, and MDA/MPO levels, but increased SOD levels in both LIPo and RIPo groups, possibly indicating a common mechanism between the two adaptations, especially in reducing lipid peroxidation, inhibition of neutrophil activation, and suppression of apoptosis [12]. However, other important mechanisms, yet to be discovered, may also be involved. There are other limitations to be addressed including optimization of protocols for LIPo and RIPo and the possibility that remote I/R could cause damage to the remote organs.

## 5. Conclusions

In conclusion, ischemic postconditioning* in situ* and remote ischemic postconditioning can decrease intestinal injuries caused by ischemia/reperfusion injury in a rabbit model. The data support reduced oxidative stress, inhibition of neutrophil activation, and suppression of cell apoptosis as mechanisms by which this protection occurs. The significance of the results lies in the possibility that such protective mechanisms, if confirmed, might be useful in clinical settings to prevent serious ischemic events of the small bowel.

## References

[1] Sise M. J. (2014). Acute mesenteric ischemia. *Surgical Clinics of North America*.

[2] Parks D. A., Granger D. N. (1986). Contributions of ischemia and reperfusion to mucosal lesion formation. *The American Journal of Physiology: Gastrointestinal and Liver Physiology*.

[3] Granger D. N., Hollwarth M. E., Parks D. A. (1986). Ischemia-reperfusion injury: role of oxygen-derived free radicals. *Acta Physiologica Scandinavica*.

[4] Suliburk J., Helmer K., Moore F., Mercer D. (2008). The gut in systemic inflammatory response syndrome and sepsis. Enzyme systems fighting multiple organ failure. *European Surgical Research*.

[5] Murry C. E., Jennings R. B., Reimer K. A. (1986). Preconditioning with ischemia: a delay of lethal cell injury in ischemic myocardium. *Circulation*.

[6] Zhao Z. Q., Corvera J. S., Halkos M. E. (2003). Inhibition of myocardial injury by ischemic postconditioning during reperfusion: comparison with ischemic preconditioning. *American Journal of Physiology—Heart and Circulatory Physiology*.

[7] Xing B., Chen H., Zhang M. (2008). Ischemic post-conditioning protects brain and reduces inflammation in a rat model of focal cerebral ischemia/reperfusion. *Journal of Neurochemistry*.

[8] Eldaif S. M., Deneve J. A., Wang N.-P. (2010). Attenuation of renal ischemia-reperfusion injury by postconditioning involves adenosine receptor and protein kinase C activation. *Transplant International*.

[9] Park J. W., Kang J. W., Jeon W. J., Na H. S. (2010). Postconditioning protects skeletal muscle from ischemia-reperfusion injury. *Microsurgery*.

[10] Kerendi F., Kin H., Halkos M. E. (2005). Remote postconditioning: brief renal ischemia and reperfusion applied before coronary artery reperfusion reduces myocardial infarct size via endogenous activation of adenosine receptors. *Basic Research in Cardiology*.

[11] Liu K.-X., He W., Rinne T., Liu Y., Zhao M.-Q., Wu W.-K. (2007). The effect of *Ginkgo biloba* extract (EGb 761) pretreatment on intestinal epithelial apoptosis induced by intestinal ischemia/reperfusion in rats: role of ceramide. *The American Journal of Chinese Medicine*.

[12] dos Santos C. H. M., Pontes J. C. D. V., Gomes O. M., Miiji L. N. O., Bispo M. A. F. (2009). Evaluation of ischemic postconditioning effect on mesenteric ischemia treatment: experimental study in rats. *Revista Brasileira de Cirurgia Cardiovascular*.

[13] Naito Y., Takagi T., Uchiyama K. (2002). Suppression of intestinal ischemia-reperfusion injury by a specific peroxisome proliferator-activated receptor-*γ* ligand, pioglitazone, in rats. *Redox Report*.

[14] Liu K.-X., Rinne T., He W., Wang F., Xia Z. (2007). Propofol attenuates intestinal mucosa injury induced by intestinal ischemia-reperfusion in the rat. *Canadian Journal of Anesthesia*.

[15] Hotter G., Closa D., Prados M. (1996). Intestinal preconditioning is mediated by a transient increase in nitric oxide. *Biochemical and Biophysical Research Communications*.

[16] Sola A., Hotter G., Prats N., Xaus C., Gelpi E., Roselló-Catafau J. (2000). Modification of oxidative stress in response to intestinal preconditioning. *Transplantation*.

[17] Mallick I. H., Yang W., Winslet M. C., Seifalian A. M. (2005). Ischaemic preconditioning improves microvascular perfusion and oxygenation following reperfusion injury of the intestine. *British Journal of Surgery*.

[18] Liu K.-X., Li Y.-S., Huang W.-Q. (2009). Immediate postconditioning during reperfusion attenuates intestinal injury. *Intensive Care Medicine*.

[19] Ferencz A., Takcs I., Horváth S. (2010). Examination of protective effect of ischemic postconditioning after small bowel autotransplantation. *Transplantation Proceedings*.

[20] Bretz B., Blaze C., Parry N., Kudej R. K. (2010). Ischemic postconditioning does not attenuateischemia-reperfusion injury of rabbit small intestine. *Veterinary Surgery*.

[21] Andreka G., Vertesaljai M., Szantho G. (2007). Remote ischaemic postconditioning protects the heart during acute myocardial infarction in pigs. *Heart*.

[22] Gritsopoulos G., Iliodromitis E. K., Zoga A. (2009). Remote postconditioning is more potent than classic postconditioning in reducing the infarct size in anesthetized rabbits. *Cardiovascular Drugs and Therapy*.

[23] Kadkhodaee M., Najafi A., Seifi B. (2014). Classical and remote post-conditioning effects on ischemia/reperfusion-induced acute oxidant kidney injury. *International Journal of Surgery*.

[24] Duilio C., Ambrosio G., Kuppusamy P., Dipaula A., Becker L. C., Zweier J. L. (2001). Neutrophils are primary source of O_2_ radicals during reperfusion after prolonged myocardial ischemia. *The American Journal of Physiology—Heart and Circulatory Physiology*.

[25] Kevin L. G., Camara A. K. S., Riess M. L., Novalija E., Stowe D. F. (2003). Ischemic preconditioning alters real-time measure of O_2_ radicals in intact hearts with ischemia and reperfusion. *American Journal of Physiology—Heart and Circulatory Physiology*.

[26] Köksoy C., Kuzu M. A., Ergün H., Demirpençe E., Zülfikaroglu B. (2000). Intestinal ischemia and reperfusion impairs vasomotor functions of pulmonary vascular bed. *Annals of Surgery*.

